# Ventilatory Muscle Training for Early Cardiac Rehabilitation Improved Functional Capacity and Modulated Vascular Function of Individuals Undergoing Coronary Artery Bypass Grafting: Pilot Randomized Clinical Trial

**DOI:** 10.3390/ijerph19159340

**Published:** 2022-07-30

**Authors:** Bruna Eibel, Juliana R. Marques, Thiago Dipp, Gustavo Waclawovsky, Rafael A. Marschner, Liliana C. Boll, Renato A. K. Kalil, Alexandre M. Lehnen, Allan R. K. Sales, Maria Claudia Costa Irigoyen

**Affiliations:** 1 Instituto de Cardiologia/Fundação, Universitária de Cardiologia (IC/FUC), Porto Alegre 90040-371, RS, Brazil; julianarm08@gmail.com (J.R.M.); gwsaude@yahoo.com.br (G.W.); lilianacboll@gmail.com (L.C.B.); kalil.renato@gmail.com (R.A.K.K.); amlehnen@gmail.com (A.M.L.); hipirigoyen@gmail.com (M.C.C.I.); 2 Programa de Pós-Graduação em Saúde Coletiva, Universidade do Vale do Rio dos Sinos (UNISINOS), São Leopoldo 93022-750, RS, Brazil; thdipp@hotmail.com; 3 Hospital de Clínicas de Porto Alegre (HCPA), Universidade Federal do Rio Grande do Sul (UFRGS), Porto Alegre 90035-903, RS, Brazil; rafamarschner@gmail.com; 4 Department of Surgery, Universidade Federal de Ciências da Saúde de Porto Alegre (UFCSPA), Porto Alegre 90050-170, RS, Brazil; 5 Institute DOR for Research and Education (IDOR), São Paulo 04501-000, SP, Brazil; allan.sales@idor.org; 6 Instituto do Coração (InCor), Universidade de São Paulo (USP), São Paulo 05403-000, SP, Brazil

**Keywords:** coronary artery bypass grafting, cardiac rehabilitation, functional capacity, flow-mediated dilatation

## Abstract

Background: Cardiac rehabilitation with aerobic exercises is the first strategy for nonpharmacological treatment in the postoperative period of individuals undergoing coronary artery bypass grafting (CABG) to improve functional capacity and vascular health. However, other exercise modalities remain uncertain regarding the same benefits. Objectives: Evaluation of the effect of different modalities of exercise, such as early cardiac rehabilitation on subjects submitted to CABG in the six-minute walk test (6-MWT) and on the percentage of flow-mediated dilatation (FMD) of the brachial artery. Methods: A randomized clinical trial in which 15 patients (62.7 ± 6.7 years) who underwent CABG were randomly assigned to the following groups: isometric (IG, Handgrip Jamar^®^), ventilatory muscle training (VG, PowerBreathe^®^) and control (CG, conventional respiratory and motor physiotherapy). All patients were attended to physically twice a day (20 min/session) for a consecutive week after the CABG (hospital admission). Functional capacity was assessed by 6-MWT and endothelial function was assessed through the technique of FMD, before and after (~7 days) admission to CABG. The doppler ultrasound videos were analyzed by Cardiovascular Suite^®^ software (Quipu, Pisa, Italy) to measure %FMD. Statistics: Generalized estimation equation, followed by Bonferroni post hoc (*p* < 0.05). Results: Systolic, diastolic and mean arterial pressure (SBP/DBP/MAP, respectively) were 133, 76 and 95 mmHg. The groups presented walking meters (m) distance before and after intervention of: IG_basal_ 357.80 ± 47.15 m vs. IG_post_ 306.20 ± 61.63 m, *p* = 0.401 (+51 m); VG_basal_ 261.50 ± 19.91 m vs. VG_post_ 300.75 ± 26.29 m, *p* = 0.052 (+39 m); CG _basal_ 487.83 ± 83.23 m vs. CG_post_ 318.00 ± 31.08, *p* = 0.006 (−169 m). %FMD before and after intervention was IG_basal_ 10.4 ± 4.8% vs. IG_post_ 2.8 ± 2.5%, *p* = 0.152; VG_basal_ 9.8 ± 5.1% vs. VG_post_ 11.0 ± 6.1%, *p* = 0.825; CG_basal_ 9.2 ± 15.8% vs. CG_post_ 2.7 ± 2.6%, *p* = 0.710 and resting mean basal blood flow was IG_basal_ 162.0 ± 55.0 mL/min vs. IG_post_ 129.9 ± 63.7 mL/min, *p* = 0.662; VG_basal_ 83.74 ± 12.4 mL/min vs. VG_post_ 58.7 ± 17.1 mL/min, *p* = 0.041; CG_basal_ 375.6 ± 183.7 mL/min vs. CG_post_ 192.8 ± 115.0 mL/min, *p* = 0.459. Conclusions: Ventilatory muscle training for early cardiac rehabilitation improved acute functional capacity and modulated mean flow of individuals undergoing CABG.

## 1. Introduction

Major surgeries, such as coronary artery bypass grafting (CABG), directly affect inspiratory muscle strength and peripheral muscle strength [1]. Cardiopneumofunctional rehabilitation is routinely prescribed, with the expectation of reducing postoperative complications and hospital stay, as well as reducing mortality rates resulting from surgery [2].

In the routine of hospital physiotherapy, the main objective is to reduce respiratory damage caused by the surgical procedure and mechanical ventilation, such as treating consolidations, atelectasis and pleural effusion, using bronchial hygiene and re-expanding techniques, in addition to tracheal aspiration, if necessary [3,4]. Another associated approach is to reestablish the patient’s confidence to sit, walk and return to routine at hospital discharge, respecting pain, reductions in peripheral muscle strength and ventilatory limitation caused by sternotomy [4].

The core of the present problematization, following the treatment recommendations for CABG, is to consider the inflammatory profile, oxidative stress and endothelial dysfunction arising from coronary artery disease (CAD) in these patients, the ventilatory dysfunction arising from CABG and the functional deconditioning arising from CAD and CABG, characterizing the frailty syndrome [2]. We propose two nonstandard therapeutic treatments in the hospital cardiac rehabilitation routine, isometric handgrip resistance exercise and ventilatory muscle training.

In this scenario, the present study was conducted to evaluate the effects of a 7-day postoperative cardiopneumofunctional rehabilitation program on functional capacity and endothelial function after CABG.

## 2. Methods

### 2.1. Study Design and Sample

We conducted a pilot randomized clinical trial designed by Consort Statement [5], approved by the Research Ethics Review Committee of Instituto de Cardiologia do Rio Grande do Sul/Fundação Universitária de Cardiologia (IC/FUC), Porto Alegre, Brazil and registered in clinicaltrials.gov NCT03096158. All participants signed a free informed consent form in compliance with resolution 466/12.

Fifteen patients who underwent CABG were randomly assigned to the following groups: (i) isometric (IG, Handgrip Jamar^®^, *n* = 5), (ii) ventilatory muscle training (VG, PowerBreathe^®^, *n* = 4); (iii) a control (CG, conventional respiratory and motor physiotherapy, *n* = 6). Randomization was performed through randomization.com with a coded distribution of a 1:1 ratio with 3 blocks of 10 allocations, accessed on 6 March 2017. The patients were blinded to allocation groups during the baseline tests.

All patients were attended to physically twice a day (20 min/session) for a consecutive week after the CABG (hospital admission), starting 24 h after extubation. The inclusion criteria comprised left-ventricular ejection fraction (LVEF) > 40%; CABG by ischemic event; not presenting other cardiopathies or angina pectoris; age between 50 to 75 years; and signing the acceptance consent ethics form.

### 2.2. Assessments

All assessments were performed in the same shift and average time, in the mornings.

#### 2.2.1. Six-Minute Walk Test

The 6-MWT used to assess functional capacity followed the guidelines proposed by the American Thoracic Society [6].

#### 2.2.2. Brachial Artery Flow-Mediated Dilatation (FMD)

Participants reported to the temperature-controlled (20 °C to 22 °C) laboratory on two occasions for FMD assessment. In preparation, participants abstained from strenuous exercise for 24 h and alcohol for 8 h as well as from any food/caffeine/stimulants for 6 h before reporting to the laboratory. Following 20 min of supine rest, brachial artery diameter and mean blood velocity were recorded via high-resolution duplex-Doppler ultrasound (Esaote, Genova, Italy) with a 7.5 to 12-MHz linear array probe. B-mode images were obtained and optimized, and Doppler velocity was recorded simultaneously. Expert-consensus protocol guidelines were followed for the performance of the FMD [7]. Briefly, after 1 min of baseline diameter and mean blood velocity measurement, an occlusion cuff, connected to an inflator, placed distal to the olecranon process, was inflated to a suprasystolic pressure (>200 mmHg) for 5 min. Brachial artery diameter and mean velocity recordings were resumed 30 s before cuff deflation, and reactive hyperemia was recorded for a further 3 min after cuff deflation. All evaluations were performed by a single experienced evaluator.

All %FMD data were analyzed using a specialized custom-designed edge-detection and wall-tracking software, Cardiovascular Suite^®^. This software tracks the vessel walls and blood velocity trace in B-mode frames via pixel density and frequency distribution algorithm. An optimal region of interest to be analyzed was selected by the sonographer, chosen based on image quality, with a clear distinction between the artery walls and lumen. The FMD was defined as the percentage change in artery diameter from baseline to the peak captured during the 3 min following cuff release. The software automatically calculated the relative diameter change and time to peak (following cuff release). Despite the initial region of interest selection being operator determined, the remaining analysis was automated and independent of operator bias [8].

### 2.3. Data Analysis

Mean blood flow was calculated from continuous diameter and mean blood velocity recordings at each of the experimental time points using the following expression: 3.14 × (diameter/2)^2^ × mean blood velocity × 60. Brachial artery conductance and resistance at rest were calculated for the equations (Mean blood flow/mean blood pressure) × 100 and mean blood pressure/blood flow, respectively. Brachial artery mean blood flow and blood velocity responses to cuff release were calculated for microvascular dilator function. These calculations encompassed the entire hyperemic response (i.e., to the point at which hemodynamics returned to resting values). Areas under the curve (AUC) for mean blood flow and blood velocity were also calculated for the entire period after cuff release using the sum of trapezoid methods. For macrovascular dilator function, the percentage change of brachial artery FMD was calculated using the following equation: %FMD = (peak diameter − base diameter)/base diameter × 100. Shear rate was defined as 4 × mean blood velocity/diameter. Shear rate AUC up to peak diameter was calculated as the stimulus for FMD. 

### 2.4. Oxidative Stress Analysis

#### 2.4.1. Biochemical Analysis

A venous blood sample was collected before and after (~7 days) admission to CABG. After centrifugation at 1.000 rpm for 10 min at 4 °C, the plasma was separated and stored at −80 °C until assayed.

#### 2.4.2. Carbonyl Measurements

The carbonyl measurements, duplicate aliquots of plasma (containing ~0.3 mgof protein) from each sample were incubated with 500 μL of 10 mM 2.4-dinitrophenylhydrazine or 1.0 mL of 2 M HCl (blank tube). After 30 min, 250 μL of 50% trichloroacetic acid was added to the aliquots. The samples were subsequently centrifuged at 8000× *g* for 30 min to obtain the protein pellets, which were immediately washed with ethanol-ethyl acetate at a 1:1 (*v*/*v*) ratio. The final protein pellets were resuspended in 500 μL of 8 M urea buffer and incubated at 50 °C for 90 min. The difference between the 2.4-dinitrophenylhydrazine-treated and HCl-treated samples (blank) was used to calculate the carbonyl content determined at 370 nm. Carbonyl content was calculated using the millimolar absorption coefficient of hydrazine (e370 nm = 21,000,000 M^−1^ cm^−1^), and the results were expressed in nmol carbonyl/mg protein [9].

#### 2.4.3. Sulfhydryl Content

Sulfhydryl content measurement was performed according to the method of Aksenov and Markesbery, where the reduction of 5,5′-dithio-bis (2-nitrobenzoic acid) (DTNB) by thiols generates a yellow derivative (TNB) whose absorption is measured spectrophotometrically at 412 nm. In brief, 30 μL of 0.1 mM DTNB was added to 120 μL of plasma. This was followed by 30 min incubation at room temperature in a dark room. Results were calculated as nmol of TNB/mg of protein [9].

##### Nonenzymatic Antioxidant Defenses

GSH levels were measured according to a standard method. Briefly, proteins were precipitated by adding sodium metaphosphoric acid for a final ratio of 1:1. Samples were centrifuged for 10 min at 7000× *g*. Fifteen microliters of tissue preparation were incubated with an equal volume of ophthaldialdehyde (1 mg/mL methanol) at room temperature for 15 min in the presence of 20 volumes (1:20, *v*/*v*) of 100 mM sodium phosphate buffer, pH 8.0, containing 5 mM EDTA. Fluorescence was measured using excitation and emission wavelengths of 350 nm and 420 nm, respectively. A calibration curve was generated using standard GSH (0.001–0.1 mM), and GSH concentrations were calculated as nmol/mg protein. GSSG levels were determined using the enzymatic recycling method described previously (Teare, Punchard et al. 1993), with some modifications. Briefly, plasma was homogenized in 4 (*w*/*v*) volumes of a sulfosalicylic acid solution (11%) and Triton X-100 (0.11%), (1:1 ratio). After incubating for 5 min at 4 °C with continuous shaking, the samples were centrifuged at 10,000× *g* for 10 min (4 °C) and the supernatant was collected for analyses of glutathione levels. For GSSG measurement, 10 μL of the supernatant were added to 110 μL of a GSH masking buffer (100 mM phosphate buffer, 1 mM EDTA, 1.1% 2-vinylpyridine), pH 7.4, and incubated for 1 h at room temperature. The samples prepared for GSSG measurement were subject ted to enzymatic analysis in a recycling buffer system containing 300 μM NADPH, 225 μM DTNB, 1.6 U/mL GR and 1.0 mM EDTA in 100 mM phosphate buffer (pH 7.4). The linear increase in absorbance at 405 nm over time was monitored using a microplate reader (SpectraMax M5, Molecular Devices, California, US). A standard curve was generated using known amounts of GSH (100 µM) and GSSG (3.47, 6.95, 13.89 uM) [10].

### 2.5. Interventions

#### 2.5.1. Isometric Handgrip Resistance Exercise

The enrolled patients performed an isometric handgrip for 20 min, once a day, 7 days per week, for 1 week until hospital discharge. An analog handgrip device (Jamar^®^) was held while sitting upright in a chair with feet flat on the floor and a single maximal contraction of the hand flexor muscles with each hand was completed to determine maximum voluntary contraction (MVC). Over a 20 min period, patients performed 2 min alternating bilateral contractions of the hand flexor muscles at 30% MVC with a 1 min rest between contractions [11]. Subjects were provided feedback and encouragement to sustain 30% MVC. In the afternoon, they performed 20 min of conventional respiratory and motor physiotherapy.

#### 2.5.2. Ventilatory Muscle Training

Inspiratory muscle function testing was performed using a pressure transducer (MVD-300, Globalmed, Porto Alegre, Brazil), connected to a system with two unidirectional valves (DHD Inspiratory Muscle Trainer, Chicago, IL, USA). Maximal static inspiratory pressure (MIP) was determined in deep inspiration from residual volume against an occluded airway with a minor air leak (2 mm). The highest pressure of six measurements (with less than 5% difference) was used to define MIP [12]. 

The enrolled patients performed ventilatory muscle training for 20 min, once a day, 7 days per week, for 1 week until hospital discharge, using the PowerBreathe^®^. During training, subjects were instructed to maintain diaphragmatic breathing at a rate at of 15 to 20 breaths/min. The inspiratory load was set at 30% of maximal static inspiratory pressure (MIP), and the training loads were adjusted to maintain 30% of MIP during the entire protocol period [12]. In the afternoon, conventional respiratory and motor physiotherapy were performed for 20 min.

#### 2.5.3. Conventional Respiratory and Motor Physiotherapy

The enrolled patients performed conventional physiotherapy for 20 min, twice a day (morning and afternoon), 7 days per week, for 1 week until hospital discharge. Our proposed hospital protocol was as follows: respiratory exercises (vibrocompression, passive manual expiratory therapy, fractional inspiration in times, diaphragmatic breathing), motor active exercises, stationary walking, walking, stairs training, respecting individual progression in days.

#### 2.5.4. Sample Size

Previous calculated sample was based on the primary outcome, functional capacity by 6-MWT. Considering a mean change of 20 m on this variable for an α error of 0.05 and power of 80%, the calculated sample size was 30 subjects—10 patients per group [1]. The effect of sample size calculated by partial eta squared was 0.059, representing medium effect.

#### 2.5.5. Statistical Analysis

Preliminary analyses were conducted using the Statistical Package for Social Sciences (SPSS, version 26, IBM, Chicago, IL, USA). Shapiro–Wilk tests were used to determine the variable distribution. The baseline comparisons between groups were performed by one-way ANOVA followed by Tukey post hoc (parametric variables) and by Pearson chi-square test (categorical variables). Parametric data were described as means and standard deviations, and nonparametric continuous data were described as medians and interquartile ranges. Generalized estimation equation (GEE) was performed to compare baseline and post between groups, followed by Bonferroni post hoc test, and the data were described as means and standard error. The statistical significance level was set at *p* < 0.05.

## 3. Results

**Figure 1:**
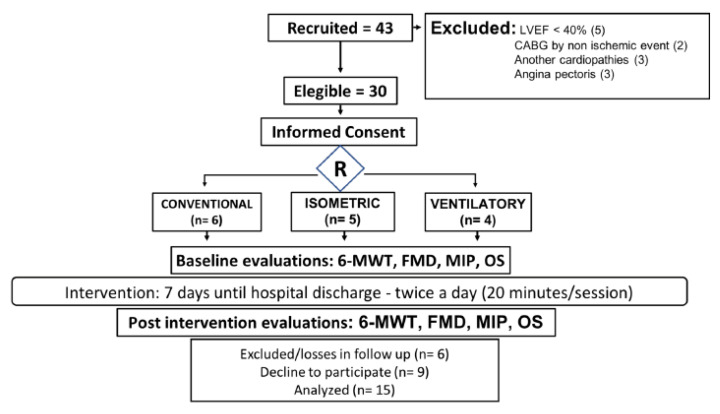
Flowchart of recruitment. Left-ventricular ejection fraction (LVEF); coronary artery bypass grafting (CABG); six-minute walk test (6-MWT); flow-mediated dilatation (FMD); maximal inspiratory pressure (MIP); oxidative stress (OS).

A total of fifteen patients completed the protocol and a total of fifteen dropped out; the flowchart of recruitment is available in Figure 1. Table 1 shows the main characteristic of patients and medication in use. Regarding blood pressure, systolic (SBP) and diastolic (DBP) arterial pressure were 133 ± 4.9 and 76 ± 3 mmHg, respectively. Regarding maximal inspiratory and expiratory pressures (MIP and MEP), as postoperative success data, we found 73.13 ± 10 and 92.5 ± 9.6 cmH_2_O at baseline analysis.

### 3.1. Functional Capacity

**Figure 2:**
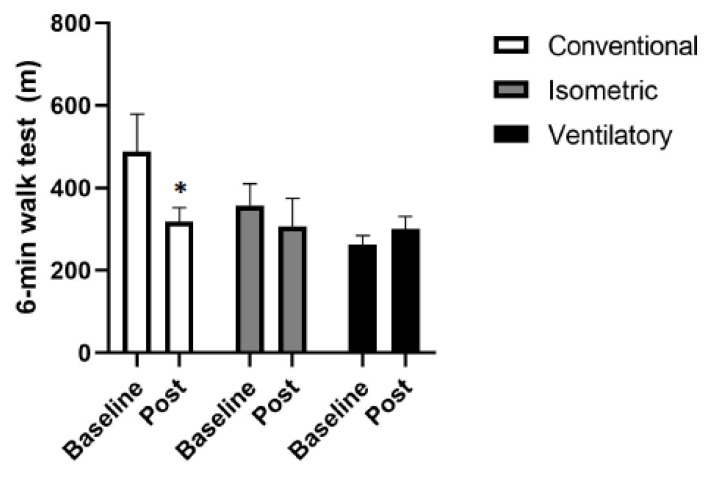
Functional capacity. Six-minute walk test (6-MWT); m: meters; * *p* < 0.05, significantly different from baseline.

The groups presented meters walked distance (m) in the 6MWT before and after intervention of IG_basal_ 357.80 ± 47.15 m vs. IG_post_ 306.20 ± 61.63 m *p* = 0.401; VG_basal_ 261.50 ± 19.91 m vs. VG_post_ 300.75 ± 26.29 m *p* = 0.052; CG_basal_ 487.83 ± 83.23 m vs. CG_post_ 318.00 ± 31.08 m *p* = 0.006 (Figure 2).

### 3.2. Endothelial Function

Values of baseline and FMD diameters did not change significantly; FMD (%) did not show a significant increase in all groups. %FMD before and after intervention was IG_basal_ 10.4 ± 4.8% vs. IG_post_ 2.8 ± 2.5% *p* = 0.152; VG_basal_ 9.8 ± 5.1% vs. VG_post_ 11.0 ± 6.1% *p* = 0.825; CG_basal_ 9.2 ± 15.8% vs. CG_post_ 2.7 ± 2.6% *p* = 0.710 and mean blood flow was IG_basal_ 162.0 ± 55.0 mL/min vs. IG_post_ 129.9 ± 63.7 mL/min *p* = 0.662; VG_basal_ 83.74 ± 12.4 mL/min vs. VG_post_ 58.7 ± 17.1 mL/min *p* = 0.04; CG_basal_ 375.6 ± 183.7 mL/min vs. CG_post_ 192.8 ± 115.0 mL/min *p* = 0.459. Shear stress of FMD had not shown any significant change. These results are available in Table 2. 

### 3.3. Oxidative Stress

**Figure 3:**
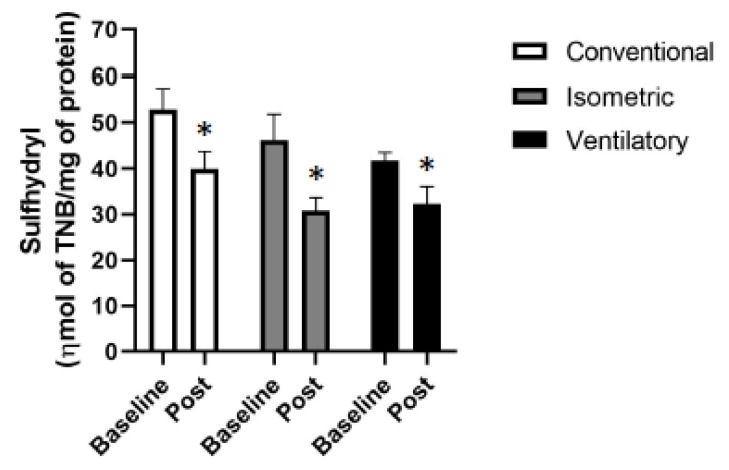
Oxidative stress-sulfhydryl. Sulfhydryl: ηmol of total bound nitrogen (TBN)/mg of protein; * *p* < 0.05, significantly different from baseline.

All the groups presented a decrease in sulfhydryl levels when compared with their respective baseline (Table 3 and Figure 3). It is also possible to observe that the conventional group had the highest values of carbonyl when compared with the others. Moreover, the other oxidative stress variables analyzed did not demonstrate any difference between the groups or between the baseline moment and after the intervention in each group (Table 3).

## 4. Discussion

This pilot randomized clinical trial shows that in stable patients with CAD submitted to CABG, a 7-day program with isometric handgrip resistance exercise and ventilatory muscle training as an addition to conventional rehabilitation attenuated the general incapacity expected in the postoperative period, with improved recovery of functional capacity since these techniques are simple, low-cost and can be implemented in the hospital routine. Considering that the baseline characteristics of the groups were similar and all subjects underwent CABG, it seems to us that an approach in which these individuals were encouraged to exercise may have had a marked impact on post discharge functional capacity [13].

Minet et al. (2012) showed that 6-MWT seems to be a predictor of endothelial dysregulation [14]. In this way, the VG was the only one that had an increase in the number of meters walked after the intervention, with concomitant reductions in oxidative stress markers, but it appears that reductions in oxidative stress markers do not improve FMD, nor other vascular measures [15,16]. 

Thereby, performing ventilatory muscle training after CABG showed improvement of functional capacity and oxidative stress (sulfhydryl) at the end of 7 days, but not global endothelial function; this can be explained by the systemic inflammatory profile of these patients and increase in peripheral vascular resistance with reduction in peripheral blood flow, requiring further follow-up to verify modulation [15,17,18,19]. In addition, we consider that medication or even antioxidants (including diet at hospital) could have affected oxidative markers in the study, considering that all conditions had reductions [18].

Further, considering that the IG utilized the muscle mass and arterial bed that was tested during FMD, increases in mean blood flow in this group make sense, being tested by FMD 24 h after the last session, equally to the other groups [8].

It is very important to highlight the response of the intervention groups (IG and VG) on functional capacity and maintenance in meters walked compared to the conventional group, demonstrating improvement in peripheral muscle and cardiopulmonary capacity [14]. Demonstrating safety regarding the alternative proposed methods, compared to the conventional group [1], these (isometric and ventilatory) may be used in routine hospital practice.

We acknowledge that the current study presented limitations due to sample size; however, we demonstrated that our effect size is representative for our outcome-functional capacity, vascular damage and oxidative stress profile, considering that this is a sample that is difficult to access in order to perform exercise due to surgical complexity.

Another issue is the use of different medications due to the complex ischemic heart disease in question, which may impact vascular modulation and oxidative stress, even the routine of patients in the postoperative period following a rigid scale of drug administration, at controlled times and doses.

## 5. Conclusions

Finally, we speculate that ventilatory muscle training for early cardiac rehabilitation can be used to improve functional capacity and modulate vascular function of patients undergoing CABG.

## Figures and Tables

**Table 1:** Demographic characteristics of the participants.

Characteristics	Conventional (*n* = 6)	Isometric (*n* = 5)	Ventilatory (*n* = 4)	*p*
Age (years) ^§^	58.8 ± 7.7	66.4 ± 4.3	63.7 ± 5.7	0.172
Male ^†^	6 (100%)	4 (80%)	2 (50%)	0.153
SPB (mmHg) ^§^	132 ± 18	136 ± 23	129 ± 20	0.213
DBP (mmHg) ^§^	78 ± 12	74 ± 14	76 ± 14	0.371
MIP	87 ± 55	73 ± 24	51 ± 8	0.047
**Comorbidities**				
T2DM ^†^	0 (0%)	1 (20%)	2 (50%)	0.153
Obesity ^†^	1 (16.7%)	1 (20%)	1 (25%)	0.949
Smoking ^†^	0 (0%)	1 (20%)	0 (0%)	0.343
Sedentary lifestyle ^†^	6 (100%)	5 (100%)	4 (100%)	- ^a^
**Cardiovascular disease**				
MI ^†^	6 (100%)	5 (100%)	4 (100%)	- ^a^
HT ^†^	2 (33.3%)	3 (60%)	3 (75%)	0.405
Hemorrhagic stroke ^†^	0 (0%)	0 (0%)	1 (25%)	0.229
**Drugs**				
β-blockers ^†^	5 (83.3%)	4 (80%)	2 (50%)	0.464
Statins ^†^	6 (100%)	5 (100%)	4 (100%)	- ^a^
ACE inhibitors ^†^	2 (33.3%)	3 (60%)	3 (75%)	0.405
Antiplatelet agents ^†^	6 (100%)	5 (100%)	4 (100%)	- ^a^
Antianginal agentes ^†^	6 (100%)	5 (100%)	4 (100%)	- ^a^
Diuretics ^†^	4 (66.7%)	4 (80%)	3 (75%)	0.880
Antidiabetics ^†^	0 (0%)	1 (20%)	2 (50%)	0.153
ARBs ^†^	2 (33.3%)	3 (60%)	3 (75%)	0.405
Anticoagulants ^†^	6 (100%)	5 (100%)	4 (100%)	- ^a^

^§^ variable described as mean ± standard deviation (SD); ^†^ variable described as frequency; SBP: systolic blood pressure; DBP: diastolic blood pressure; MIP: maximal inspiratory pressure; T2DM: Type 2 diabetes mellitus; MI: acute myocardial infarction; HT: systemic hypertension; ACE: angiotensin-converting enzyme; ARBs: angiotensin II receptor blockers. ^a^ No statistics because the variable is a constant.

**Table 2:** Flow-mediated dilatation of brachial artery (baseline) preintervention and (post) postintervention in patients undergoing coronary artery bypass grafting.

	Conventional (*n* = 6)			Isometric (*n* = 5)			Ventilatory (*n* = 4)		
	Baseline	Post	*p*	Baseline	Post	*p*	Baseline	Post	*p*
**FMD** (%)	9.2 ± 15.8	2.7 ± 2.6	0.710	10.4 ± 4.8	2.9 ± 2.5	0.152	9.8 ± 5.1	11.0 ± 6.1	0.825
**Baseline brachial diameter** (mm)	5.39 ± 1.19	4.86 ± 0.40	0.696	4.48 ± 0.08	4.51 ± 0.20	0.842	4.20 ± 0.35	4.16 ± 0.32	0.800
**Peak brachial diameter** (mm)	4.95 ± 0.29	4.94 ± 0.32	0.994	4.96 ± 0.30	4.66 ± 0.30	0.235	4.57 ± 0.31	4.58 ± 0.34	0.866
**Resting mean blood flow** (mL/min)	375.6 ± 183.7	192.8 ± 115.0	0.459	162.0 ± 55.0	129.9 ± 63.7	0.662	83.74 ± 12.4	58.7 ± 17.1	0.041
**Resting shear rate** (s^−1^)	87.3 ± 30.7	84.6 ± 36.1	0.932	166.6 ± 58.9	118.9 ± 62.0	0.564	98.5 ± 16.2	60.6 ± 17.1	0.072
**Mean blood flow AUC** (mL/min)	324.83 ± 115.7	371.78 ± 158.46	0.658	253.27 ± 15.59	348.08 ± 43.42 ^a^	0.103	413.76 ± 226.45	185.31 ± 24.42	0.341
**Shear rate AUC** (s^−1^)	292.2 ± 91.2	209.0 ± 43.0	0.232	241.5 ± 15.4	375.5 ± 58.3	0.003	217.9 ± 55.9	252.9 ± 79.6	0.495

FMD: flow-mediated dilatation; mean blood flow AUC: mean blood flow of area under curve (blood flow AUC). Data represent the mean ± SEM and the analyses were by GEE followed by Bonferroni post hoc test. ^a^ *p* = 0.003, significantly different compared to ventilatory group.

**Table 3:** Oxidative stress analysis.

	Conventional (*n* = 6)			Isometric (*n* = 5)			Ventilatory (*n* = 4)		
	Baseline	Post	*p*	Baseline	Post	*p*	Baseline	Post	*p*
**Carbonyl** (ηmol carbonyl/mg protein)	10.4 ± 2.05	8.7 ± 0.907	0.449	0.816 ± 0.087 ^b^	1.05 ± 0.311 ^b^	0.361	1.23 ± 0.248 ^b^	0.864 ± 0.227 ^b^	0.372
**GSH** (ηmol/mg protein)	18.9 ± 0.087	19.3 ± 0.183	0.119	19.0 ± 0.036	19.1 ± 0.040	0.198	18.3 ± 0.533	19.0 ± 0.047	0.130
**GSSG** (ηmol/mg protein)	0.184 ± 0.003	0.196 ± 0.007	0.120	0.185 ± 0.001	0.188 ± 0.002	0.176	0.158 ± 0.021	0.187 ± 0.002	0.132
**GSH**/**GSSG**	103.4 ± 1.39	98.7 ± 2.32	0.095	102.5 ± 0.58	101.4 ± 0.61	0.197	125.9 ± 18.74	101.9 ± 0.74	0.189
**Sulfhydryl** (ηmol of TNB/mg of protein)	53.0 ± 4.07	39.9 ± 3.48	0.009	46.2 ± 5.11	30.8 ± 2.56	0.021	41.7 ± 1.48 ^a^	32.3 ± 3.28	<0.001

GSH: reduced glutathione; GSSG: oxidized glutathione. Data represent the mean ± SEM and the analyses were by GEE followed by Bonferroni post hoc test. ^a^ *p* = 0.029; ^b^ *p* < 0.001 compared with conventional group.

## Data Availability

All authors take responsibility for all aspects of the reliability and freedom from bias of the data presented and their discussed interpretation.
